# Prevalence and risk factors of osteoarthritis in patients at a public hospital in Limpopo province

**DOI:** 10.4102/safp.v66i1.5966

**Published:** 2024-11-25

**Authors:** Tsundzuka Masangu, Boikhutso Tlou, Thembelihle Dlungwane

**Affiliations:** 1 Department of Public Health Medicine, Faculty of Health Sciences, University of KwaZulu-Natal, Durban, South Africa

**Keywords:** prevalence, risk factors, osteoarthritis, public hospital, Limpopo, South Africa

## Abstract

**Background:**

Osteoarthritis (OA) is a public health concern affecting millions globally. Osteoarthritis has been ranked as the 12th leading cause of disability among the ageing population globally. In addition, OA can lead to disability, which can affect the quality of life and physical and emotional well-being.

**Methods:**

A cross-sectional survey was conducted. An interviewer-administered questionnaire was utilised. Logistic regression was used to identify OA-related factors in the univariable and multivariable models. A *p*-value less than 0.05 was deemed statistically significant.

**Results:**

A total of 210 participants responded to the questionnaire. The overall prevalence of OA at the public hospital was 55.7% in adults over 18 years willing to participate. Among the study participants, females, individuals over the age of 50 years, and obese patients reported a high prevalence of OA. Family history and knee and hip pain were significantly associated with OA (*p* < 0.05). Participants with a family history of OA were 6.9 times more likely to have OA, those with knee pain were 22.8 times more likely and those with hip pain were 5.5 times more likely after adjusting for the other variables.

**Conclusion:**

A high proportion of patients reported to have OA. Family history, knee pain and hip pain were strongly associated with OA. Understanding the prevalence and risk factors associated with OA is crucial for developing targeted interventions for prevention and management.

**Contribution:**

Targeted health promotion and education interventions are needed for prevention and early management.

## Introduction

Osteoarthritis (OA) is one of the most common joint disorders worldwide.^1^ Osteoarthritis is the leading cause of disability among the ageing population, affecting 3.3% – 3.6% of the population globally, which amounts to approximately 237 million people.^2,3^ Osteoarthritis is a heterogeneous disease characterised by multi-tissue failure in diarthrodial joints.^2^ This results in pain, swelling and stiffness, leading to the inability to move.^4,5^ The frequently affected areas are hips, knees, hands, feet and spine.^4,5^

The prevalence of OA ranges from 20% to 60% globally.^1,4,6^ Regional variations in OA prevalence and risk factors have been observed, influenced by socioeconomic status, cultural practices and healthcare access.^4,6,7^ In addition, urbanisation and lifestyle changes contribute to the rising burden of OA.^1,8^ The high burden of OA affects the quality of life, mobility and independence, resulting in social isolation, curtailment of leisure and employment activities and decreased workplace productivity.^9,10^ Direct healthcare costs, indirect costs to people living with OA and intangible costs of living with a chronic debilitating condition all contribute to the economic burden.^10,11^

The prevalence of OA is expected to increase, making OA the fourth most common cause of disability worldwide.^12^ Studies have revealed several risk factors associated with OA, including gender, age, obesity, diet, occupation, level of physical activity, family history, metabolic diseases, previous joint injury and hip and knee pain.^6,7,9,13^ Understanding the prevalence and risk factors associated with OA is essential for developing targeted interventions for prevention and early management.

Treatment guidelines recommend the promotion of self-management, a healthy weight and a combination of strengthening and aerobic exercises as core management strategies.^14,15^ Knowledge of the presence of OA risk factors, especially modifiable ones, in younger populations may aid in the earlier identification of individuals at high risk of developing the condition. It may offer an opportunity to prevent or postpone its development.^16^ When non-surgical therapies fail to control OA symptoms, joint replacement is recommended.^17^

There is a paucity of literature on the prevalence and risk factors associated with OA at public hospitals in South Africa. The studies that have been conducted on risk factors and prevalence have focused on urban settings. The prevalence ranges from 20% to 60%, with disparities across the different regions globally.^4,6,7^ Limpopo province is located in the rural part of South Africa. This study sought to determine OA’s prevalence and risk factors among patients at a public hospital in Limpopo province.

## Research materials and methods

### Study design

A cross-sectional study design with an analytic component was implemented – patients attending physiotherapy who were above the age of 18 years and who gave voluntary informed consent to participate.

### Study setting

The research was conducted at a district hospital in a town in Limpopo province. The services include casualty, medicine, paediatrics, maternity, surgery, orthopaedic, dietetics and rehabilitation services. The participants were patients receiving treatment at the general physiotherapy outpatient clinic. The physiotherapy clinic runs daily and attends to approximately 30–60 patients weekly and is run by a qualified physiotherapist in the clinic. The patients are seen based on appointments.

### Study population and sampling

Non-probability sampling was used. Patients attending physiotherapy clinics at the district hospital were invited to participate. Patients who have severe neurological disorders that impact physical function and those who were previously diagnosed with other types of arthritis, for example, rheumatoid arthritis or psoriatic arthritis, were excluded. Our study had a population size of 350, and we utilised the Krejcie and Morgan sample size table to determine the appropriate sample size. According to the table, using the 95% confidence level and a margin of error of ±5%, the required sample size for a population of 350 was 181. We then accounted for a 10% non-response rate of the initial sample size, increasing our sample size to 210 study participants.

A total of 210 questionnaires were administered based on those who met the inclusion criteria, which was 210. A pilot study was conducted to ensure that the questionnaire was user-friendly. Ten participants were selected randomly for pilot testing, which was done to assess the reliability of the tool. Amendments were made to the tool based on the participant’s responses, especially to questions that were unclear to the study participants.

### Tool and data collection

An interviewer-administered questionnaire was used to collect data from February 2020 until September 2020. Experts on musculoskeletal health and orthopaedics validated the questionnaire. A qualified translator translated the questionnaire into Xitsonga. Xitsonga is the language widely spoken in the community around the hospital. The translations were back-translated into English to ensure they were compatible with the original questionnaire. The interviewer was fluent in Xitsonga, Venda and Sepedi. The questionnaire consisted of three sections. The first part covered the demographic characteristics of the participants, such as age, gender, educational level, type of dwelling (rural or urban), painful sites and body mass index (BMI). Anthropometric measures to assess nutritional status were recorded: height and weight. The BMI was calculated (weight [kg]/height [m^2^]) and used to rate the nutritional status according to World Health Organization (WHO) criteria. The WHO classifies BMI into underweight (< 18.5 kg/m^2^), normal (BMI 18.5 kg/m^2^ – 24.9 kg/m^2^), overweight (25.0 kg/m^2^ – 29.9 kg/m^2^) and obese (BMI ≥ 30 kg/m^2^). The second part consisted of the history of OA, and the last part consisted of occupational and health-related factors.

Qualified physiotherapists screened the patients, and those who met the inclusion criteria and agreed to participate were referred to the research assistant. One research assistant was hired to collect the data. The researcher trained the research assistant before data collection and administration of the tool, and they did the pilot testing together. At the time of data collection, the research assistant was in the physiotherapy department daily and interviewed those who agreed in a separate private room before their physiotherapy consultations. The questionnaires were manual and captured digitally by the researcher once completed.

### Data analysis

Data were captured onto a Microsoft Excel spreadsheet and then imported to Statistical Package of Social Sciences (SPSS) version 27. A *p*-value of less than 0.05 was deemed statistically significant. The Pearson Chi-square test and logistic regression were used to identify risk factors associated with OA in the bivariate and multivariable analysis, respectively.

### Ethical considerations

Ethics approval was obtained from the Biomedical Research Ethics Committee (BREC/00000310/2019) and the Limpopo Provincial Department of Health. Participants who agreed to participate in the study signed an informed consent form. Study participants’ information was kept confidential by not capturing codes as identifiers on questionnaires.

## Results

Most participants were females (81.0%), aged between 51 years and 70 years, with a high BMI. The prevalence of current OA among patients attending physiotherapy at the public hospital was 55.7%. Overall, knee OA (49.5%) and hip OA (26.7%) were reported to result in the most severe level of pain experienced by OA patients. Most participants in the study were obese (33.3%) and overweight (37.6%). The proportion of married individuals was 60%. Approximately 89.0% of participants were from rural areas, and only 11.0% resided in urban areas. In addition, many participants lacked educational background, particularly pre-matric (79.0%) and were generally involved in physically demanding occupations. The majority of participants were Tsonga-speaking people (94.3%), with a very low proportion of Venda-speaking people (3.3%) and Sepedi-speaking people (2.4%). The results of demographic information are summarised in Table 1.

**TABLE 1:** Socio-demographic characteristics of the study participants.

Characteristic	Frequency (*n*)	Percentage (%)
**Gender**		
Male	40	19.0
Female	170	8.01
**Age group (years)**		
< 31	8	3.8
31–50	65	30.9
51–70	100	46.6
> 71	37	17.6
**BMI**		
Underweight	3	1.4
Normal	58	27.6
Overweight	79	37.6
Obese	70	33.3
**Marital status**		
Unmarried	84	40.0
Married	126	60.0
**Educational level**		
Pre-matric	167	79.0
Post matric	43	21.0
**Geographical distribution**		
Rural	187	89.0
Urban	23	11.0
**Knee pain**		
Yes	104	49.5
No	106	50.5
**Hip pain**		
Yes	56.0	26.7
No	154	73.3

BMI, body mass index.

The prevalence of OA was also assessed. The prevalence of OA in the study findings is 55.7%, as summarised in Table 2.

**TABLE 2:** Prevalence of osteoarthritis.

OA	Frequency (*n*)	Percentage (%)
Yes	117	56.0
No	93	44.0

OA, osteoarthritis.

A bivariate analysis using the Fisher exact test and the Chi-square test was used to assess the association between OA and the list of risk factors: age, gender, BMI, hypertension, diabetes mellitus, cardiovascular diseases, knee pain, hip pain, family history, level of physical activity and joint injury. Out of the risk factors listed, only family history, knee pain and hip pain were found to be significantly associated with OA (*p* < 0.05). Participants who came from a family with a history of OA were more likely to have OA as opposed to those who came from families without a history (*p* < 0.001). In addition, knee pain was also found to be significantly associated with OA; most participants with knee pain were more likely to have OA when compared to those without knee pain (*p* < 0.001). Furthermore, hip pain was also significantly associated with OA (*p* = 0.001). Most participants with hip pain were likely to have OA compared to those with no hip pain. The majority of the participants with OA were physically active even though the association was not statistically significant (*p* = 0.1). Similarly, BMI was not significantly associated with OA even though the majority of participants who had OA were overweight and obese (*p* = 0.1). Lastly, age was not significantly associated with OA; however, most older participants had OA (*p* = 0.1). Results are summarised in Table 3.

**TABLE 3:** Bivariate association of various risk factors with the risk of osteoarthritis (*N* = 210).

Variable	Osteoarthritis				*P*
	Yes		No		
	*n*	%	*n*	%	
**Gender**	-	-	-	-	0.3
Male	21	10.0	22	10.4	-
Female	96	45.7	71	33.8	-
**Age (years)**	-	-	-	-	0.1
< 30	3	1.43	7	3.3	-
31–50	26	12.7	30	14.3	-
51–70	62	29.5	42	20.0	-
> 71	26	12.4	14	6.7	-
**BMI**	-	-	-	-	0.1
Underweight	0	0.0	3	1.4	-
Normal	31	14.8	27	12.9	-
Overweight	42	20.0	37	17.6	-
Obese	44	30.0	26	12.4	-
**Hypertension**	-	-	-	-	0.6
Yes	76	36.2	57	27.1	-
No	41	19.5	36	27.1	-
**Diabetes mellitus**	-	-	-	-	0.2
Yes	13	6.2	6	2.9	-
No	104	49.5	87	41.2	-
**Cardiovascular**	-	-	-	-	0.2
Yes	7	3.3	2	0.9	-
No	110	52.4	91	43.3	-
**Family history**	-	-	-	-	< 0.001
Yes	88	41.9	29	13.8	-
No	32	15.2	61	29.0	-
**Level of physical activity**	-	-	-	-	0.1
Low impact	27	12.9	17	8.0	-
Moderate impact	65	30.9	64	30.5	-
High impact	25	11.9	12	5.7	-
**Knee pain**	-	-	-	-	< 0.001
Yes	89	42.4	15	7.1	-
No	28	13.3	78	37.1	-
**Hip pain**	-	-	-	-	0.001
Yes	42	20.0	14	6.7	-
No	75	35.7	79	37.6	-
**Joint injury**	-	-	-	-	0.2
Yes	14	6.7	17	8.1	-
No	103	49.0	76	36.2	-

BMI, body mass index.

After conducting the bivariate analysis described in Table 3, a multivariate analysis was performed, where all the variables that were found to be statistically significant to OA in the bivariate analysis, namely, family history, knee pain and hip pain were put into a final multivariate model as shown in Table 4.

**TABLE 4:** Logistic regression analysis for risk factors associated with osteoarthritis.

Variables	Adjusted odds ratio (AOR)	95% CI for OR		*P*
		Lower	Upper	
**Family history**	-	-	-	< 0.001
No	1	-	-	-
Yes	6.9	3.0	14.7	-
**Knee pain**	-	-	-	< 0.001
No	1	-	-	-
Yes	22.8	9.8	53.0	-
**Hip pain**	-	-	-	< 0.001
No	1	-	-	-
Yes	5.5	2.2	13.9	-

CI, confidence interval; OR, odds ratio.

Firstly, participants who came from a family with a history of OA were 6.9 times more likely to have the condition as compared to participants who came from families without a history after adjusting for knee pain and hip pain. Secondly, participants with knee pain were 22.8 times more likely to have OA as compared to participants without knee pain after adjusting for family history and hip pain. Thirdly, participants with hip pain were 5.5 times more likely to have OA as compared to participants without hip pain after adjusting for family history and knee pain. Results are summarised in Table 4.

## Discussion

This study assessed OA’s prevalence and risk factors among patients attending physiotherapy in a public health facility in Limpopo. The prevalence of OA is 55.7%, which is consistent with a study conducted among patients reporting an overall prevalence of 55% for OA in South African urban areas.^18^ The high prevalence of OA in this study could be a high number of patients who are overweight and those with knee pain.

In this study, the prevalence of OA was high among females, 45.7%, consistent with other research findings.^19,20^ Brennan-Olsen et al. made a similar observation in their meta-analysis, finding that women are more likely than men to have OA.^7^ The findings of this study concur with findings from a systematic review, which concluded that women have a higher prevalence of OA than men. However, no significant relationship between gender and OA was found in this investigation.

Participants with OA in this study had a high prevalence of obesity (33.8%) and overweight (38.1%). However, the BMI was not significantly associated with OA, despite most participants with OA being either overweight or obese (*p* = 0.1). A study conducted in Canada reported a significant association between BMI and risk of knee and hip OA and concluded that being obese (BMI > 30 kg/m^2^) was significantly associated with the prevalence of knee (OR: 4.37; 95% CI: 2.08, 9.20) and hip (OR: 2.52; 95% CI: 1.17, 5.43) OA.^19^ The impact of BMI may not be just biomechanical but may also have some metabolic and inflammatory systemic effects.^20^ Further research needs to be conducted to get more insight on these findings in our context.

A high proportion of participants reported having OA in the age group 51–70 years (29.5%) in the current study. Although this study showed no significant association between age and OA, more older participants had OA (*p* = 0.1). Previous studies on the prevalence of OA have concluded that it increases with age, occurring after age 40–50 years.^6,7^ Within this group of participants, age was not associated with the severity level. While insignificant, the trend was for older knee OA participants who reported tolerable pain. This contrasts with data from studies where respondents were asked to rate their severity of OA as mild, moderate or severe, where increasing age was associated with increased self-reported severity.^5,6,12^ More research is needed to ascertain the differences observed in the current study.

Knee OA is an imprecise guide to the likelihood that knee pain or disability will be present.^14^ In this study, the percentage of participants who responded ‘yes’ to having knee or hip pain yielded a higher prevalence for both knee and hip pain. In this study, knee pain was significantly associated with OA (*p* < 0.001) when participants were asked if they had had knee pain in the past 6 months or a year. Hip pain was also significantly associated with OA (*p* = 0.001). This is consistent with other studies that reported that hip and knee pain is usually related to OA.^16,21,22,23^

Familial aggregation of OA has long been recognised, indicating a potential genetic component in the aetiology of the disease.^24^ Numerous studies have explored the association between family history and OA, shedding light on the genetic predisposition to this condition.^6,25,26^ In the current research, family history was significantly associated with OA (*p* < 0.001). The findings concur with a recent study conducted in Egypt, indicating that family history was significantly associated with the disease risk.^26^ Another study conducted in India concluded that a family history of knee pain is strongly related to the development of OA.^25^

The findings of our study reveal significant associations between a family history of OA, knee pain, hip pain and the presence of OA. After adjusting for potential confounding variables such as knee pain and hip pain, participants with a family history of OA were 6.9 times more likely to develop OA compared to those without such a history. This suggests a strong familial predisposition to the condition, consistent with previous research.^6,16,26^ Furthermore, participants reporting knee pain exhibited a substantially increased risk of OA, being 22.8 times more likely to have the condition compared to those without knee pain, even after adjusting for family history and hip pain. This association underscores the clinical importance of knee pain as a potential indicator or precursor of OA development.^16,23,26^ Similarly, participants experiencing hip pain demonstrated a notable elevation in the likelihood of OA, being 5.5 times more likely to be affected compared to those without hip pain, following adjustment for family history and knee pain. This observation supports the notion that hip pain may serve as a significant clinical marker for the presence of OA.^16,21,22^

### Limitations of the study

The data were self-reported and, therefore, subject to information bias. The study did not include observations or physical assessments where the patient reported pain. The study was conducted in a physiotherapy clinic in a rural district hospital setting, and the results may not be generalisable to patients in other settings. Our study presents wide confidence intervals for the estimated rates. We observed considerable variability in the data, contributing to wider intervals. This variability may stem from individual differences among participants or other uncontrolled factors. Identifying and controlling for these sources of variability in future research could narrow the confidence intervals.

### Recommendation and conclusion

A high proportion of patients reported to have OA. There is a suggestion that there is an association between OA with knee pain, hip pain and family history among our participants. More research is needed to establish the prevalence of OA in other public health facilities across Limpopo province in order to make conclusions about associations of OA in Limpopo.
